# Donation After Circulatory Death Islets are Comparable to Standard-of-Care Donation After Brain Death Islets: Analysis of 801 Consecutive Human Islet Isolations

**DOI:** 10.1097/TXD.0000000000001930

**Published:** 2026-03-20

**Authors:** Meirigeng Qi, Keiko Omori, Christopher Orr, Luis Valiente, Shiela Bilbao, Amber Tucker, Jeffrey Rawson, Nelson Gonzalez, Joshua Verret, Juan Cuadra, Jacob Mares, Kevin Jou, Doreen Ligot, Bennie Balandran, Hirotake Komatsu, Miryam Mehra, Karen Ramos, Jeannette Hacker-Stratton, Ismail H. Al-Abdullah, Jeffrey S. Isenberg, Fouad Kandeel

**Affiliations:** 1 Department of Translational Research and Cellular Therapeutics, Arthur Riggs Diabetes & Metabolism Research Institute, City of Hope National Medical Center, Duarte, CA.; 2 Department of Surgery, University of California, San Francisco, San Francisco, CA.; 3 Department of Diabetes Complications & Metabolism, Arthur Riggs Diabetes & Metabolism Research Institute, City of Hope National Medical Center, Duarte, CA.

## Abstract

**Background.:**

Transplantation of isolated human islets is a valuable therapy for individuals with severe type 1 diabetes. A shortage of suitable pancreata and islets hinders access to islet transplantation (IT). Organ donation after circulatory death (DCD) islets are believed inferior and not appropriate for clinical IT. We readdressed this and compared outcomes between DCD and standard-of-care donation after brain death (DBD) islets.

**Methods.:**

The data sample comprised 801 human islet isolations >2 decades from a single IT center. Islets from DCD and DBD donor organs were compared for islet outcome and quality. In some instances, a noninferior equivalency analysis was performed.

**Results.:**

Across the study interval, islet yield, viability, and function remained high despite a deterioration in donor health biometrics. Noninferior analysis demonstrated that DCD islets were equivalent to DBD islets in islet yield post-culture and in murine diabetes reversion. In fact, in vivo islet function trended to more diabetes reversal in mice receiving DCD islets versus animals given DBD islets (75% versus 67%). As well, no significant differences were observed between groups in age, body mass index, pancreas cold ischemia time, digestion switch time, islet yield (success >250 000 islet equivalent), purity (>80%), recovery (>75%), post-culture viability, transplantable preparations, or insulin stimulation index. The median days of hospitalization and warm ischemia time were significantly greater in DCD versus the DBD group. Predictably, elevated donor hemoglobin A1c negatively impacted islet quality regardless of donation death status.

**Conclusions.:**

Results from islet isolations >2 decades from a single center are presented. DCD islets were found to be equivalent to DBD islets on several relevant metrics. These findings encourage wider use of DCD donor islets for clinical IT.

## Graphical abstract

**Figure:**
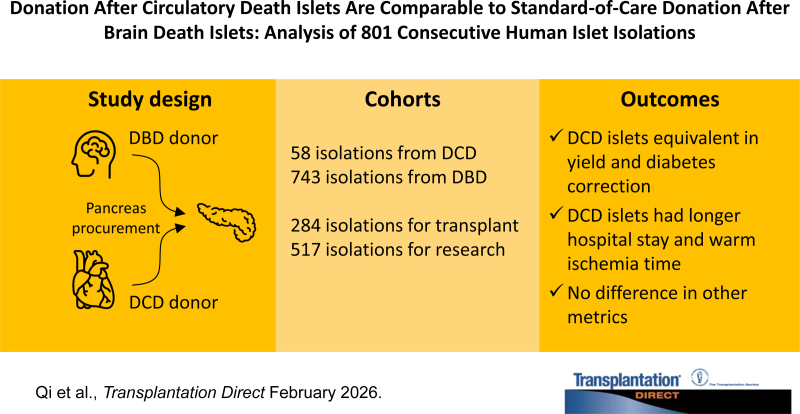

## INTRODUCTION

Clinical islet transplantation (IT) provides substantial benefits for individuals with severe type 1 diabetes (T1D).^1^ It is a practical means of restoring endogenous insulin production^2^ and of improving quality of life.^3^ From 2000 to 2022, some 4365 islet isolation procedures were carried out at 94 institutions (average 46 isolations per institution).^4^ IT relieved severe hypoglycemia in >90%, ideal blood glucose control was obtained in 60%, and 30% were insulin-independent 5 y after the procedure.^2,5,6^ A lack of quality pancreata and islets is a barrier to IT access.^7,8^ Contributing to this is the underutilization of available donor pancreata. For example, islets from donation after circulatory death (DCD) organs are used less often in clinical IT perhaps because of quality concerns.^9,10^

Clinical IT and basic research with human islets are contingent upon the efficiency of the isolation process and the quality of the final product.^11,12^ To a degree, the isolation process has been standardized.^13^ Results of islet isolation come from several sources, including the United Network for Organ Sharing^14^ and the Integrated Islet Distribution Program,^15^ as well as case series^16^ and multicenter series.^17^ Yet, islet isolation outcome data is often disparate and incomplete. There are few reports of large volume islet isolation experiences from clinically active IT programs and high-volume isolation centers. As clinical IT encompasses an element of art^18^ the detailed scrutiny of the islet isolation process remains relevant.

DCD donor organs have been underutilized for islet isolation because of poor isolation outcomes. Herein, we tested the hypothesis that DCD islets are equivalent to donation after brain death (DBD) islets. An assessment of 801 consecutive human islet isolations spanning 20 y at City of Hope National Medical Center was completed. Along all quality metrics, DCD islets matched DBD islets. On equivalency analysis of islet isolation yield and diabetes reversion in mice, DCD islets were found comparable to DBD islets. These results from a large series of human islet isolations support consideration of DCD islets for clinical IT.

## MATERIALS AND METHODS

### Study Information

Donor organs were obtained from deceased individuals. Consent was acquired by the organ procurement organization. Human data were de-identified and maintained in secure files at the Arthur Riggs Diabetes & Metabolism Research Institute of City of Hope National Medical Center (Duarte, CA). Analysis of the offered and accepted organs, cause of death, islet isolation outcomes, and quality assessment by year, with data from the isolations of each year averaged, was conducted. Equivalency (noninferiority) between DCD and DBD islets was assessed. As well, the impact of donation category, DCD versus DBD, and of donor hemoglobin A1c (HbA1c), on islet metrics and quality assays were tested. Comparison of outcomes between the isolations used for research (abbreviated Rese) and those used for transplant (abbreviated Tran) was carried out. The diabetic donor cohort islet data was excluded from this analysis as such islets would not be used for transplantation.^19^

### Islet Isolation and Quality Assay

Donor screening was by the organ acceptance team using the eligibility criteria of age, body mass index (BMI), cause of death, and medical history. Infectious disease testing and visual organ inspection preceded final acceptance. Islet isolation was approved under an Institutional Review Board protocol (IRB 01046). After staining with dithizone, the islets were counted.^20^ Functional quality of isolated islets was assessed using glucose-stimulated insulin secretion,^21,22^ oxygen consumption rate (OCR),^23^ and restoration of euglycemia in diabetic mice,^22^ as we published. Detailed methods can be found in the **Supplemental Digital Content** (**SDC**, https://links.lww.com/TXD/A846).

#### Statistics

Data in the figures and tables are presented as mean ± SEM and median (Q1–Q3; Q1 = 25th percentile and Q3 = 75th percentile). Figures were generated using GraphPad Prism 10.4.2 (GraphPad Software, La Jolla, CA). One-way ANOVA with Tukey post hoc test was used to run multiple comparisons of data from each year. Unpaired (2-tailed) nonparametric Student *t* test was performed to compare the data between islets used for research versus islets earmarked for transplantation. Kruskal-Wallis rank-sum test, Pearson chi-square test, Welch 2-sample *t* test, and Wilcoxon rank-sum test were applied as appropriate for analysis of the data. Equivalence analysis between the islets from DBD and DCD donors was performed. Islet yield and function in diabetic mice are considered predictive of clinical IT success.^24,25^ Regarding islet yield, the equivalence boundaries were defined using the reported coefficients of variance error rate based on manual islet counting.^26^ Regarding posttransplantation reversal of diabetes in mice, the equivalence boundaries were defined using the coefficients of variance from successful reversal of diabetes in mice from donor islets, which were shown to result in insulin independence in clinical IT recipients for at least 1 y following transplantation. A *P* < 0.05 was considered significant.

## RESULTS

### Human Islet Manufacturing at a Single Center

Human islet isolation at the City of Hope National Medical Center began in 2004 and islet distribution started in 2010. Between 2010 and 2013, islet isolation was paused because of protocol changes (**Figure S1**, **SDC**, https://links.lww.com/TXD/A846).

### Pancreas Organs Screened and Accepted for Islet Isolation

**FIGURE 1.**
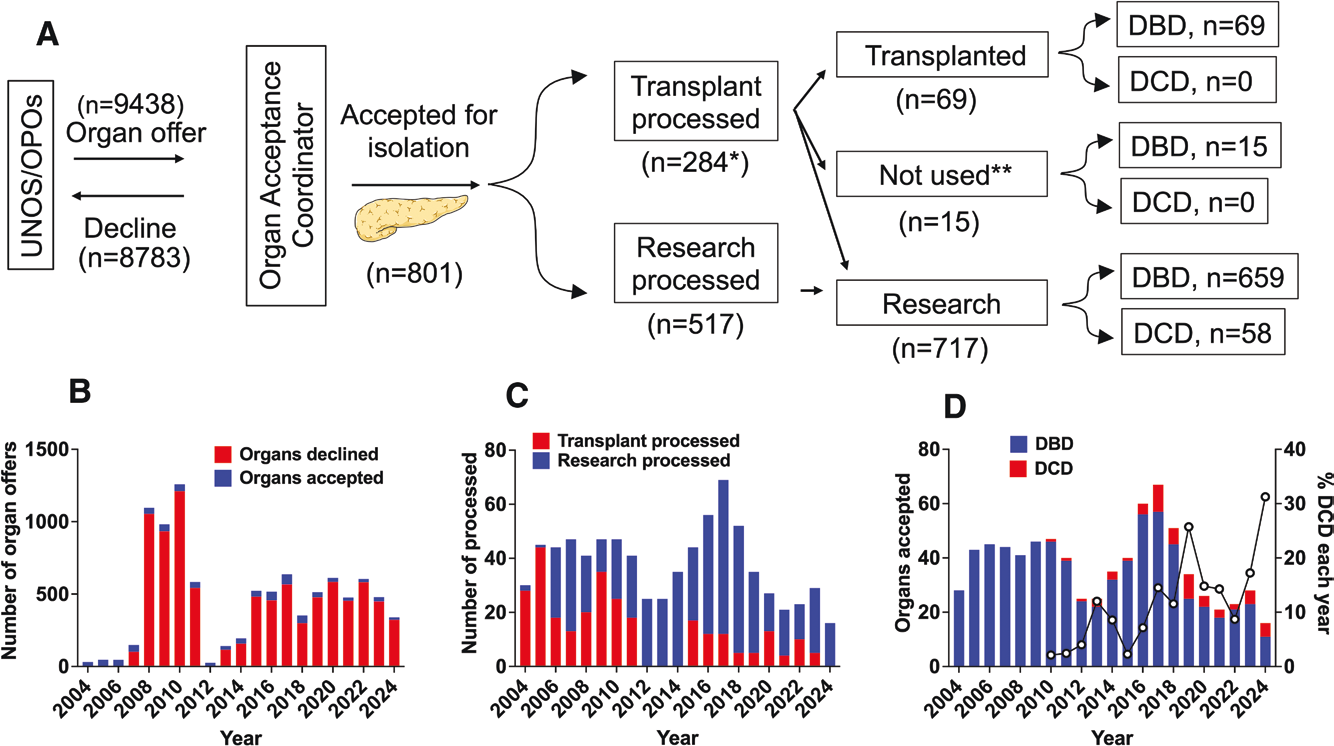
Pancreas organs screened and accepted for islet isolations from 2004 to 2024. A, The flowchart describes the human islet isolation process for usage of islets in clinical IT and research. The organ acceptance coordinator was responsible for screening pancreas donors that were offered by the United Network for Organ Sharing or by organ procurement organizations. The number of DBD and DCD donors arising at the end of the work flow are pointed out on the far right. All islet isolations were performed in a current good manufacturing practice (cGMP) facility. The same protocol was used for isolating islets for transplantation and research. *Isolations that did not meet transplant criteria were used for research. **Several islet preparations were disqualified from transplant and not used for research because of lack of research consent. B, Number of pancreata declined after screening (red bar) and accepted for islet isolation (blue bar). The number of offers screened and declined from 2004 to 2006 and 2012 was not available. C, The number of organs processed for islet isolation for transplantation (red bar) and research (blue bar). From 1012 to 2014, clinical IT was not performed. D, Number of pancreata from donation after brain death (DBD, blue bar) and donation after circulatory death (DCD, red bar). The black line in the graph shows the % of DCD donors for each year. Data on DCD organs from 2004 to 2009 were not available. DBD, donation after brain death; DCD, donation after circulatory death; IT, islet transplantation; OPO, Organ Procurement Organization; UNOS, United Network for Organ Sharing.

Organ acceptance records from 2004 to 2024 showed that 8.5% (801/9438) of organ offers were accepted (Figure 1A and B). Of these, 284 (35.4%) were processed for clinical IT and 517 (64.6%) for research (Figure 1C). All islets used in clinical IT were isolated from DBD donors (Figure 1A). Beginning in 2010, DCD organ acceptance numbers have steadily increased accounting for 8.0% (58/717) of the research islet cohort (Figure 1D). Islet processing for clinical IT declined because of reimbursement issues, regulatory changes, and fewer suitable donors and recipients (Figure 1C). Of the 284 organs processed for IT, 69 (24.3%) yielded transplant-worthy islet preparations. Fifteen preparations (5.3%) were not included because of a lack of consent (Figure 1A). Acceptance rates varied from 3.7% in 2010 to 31.8% in 2007 (Figure 1B). Organ offers increased between 2008 and 2010 because of more transplant listings (Figure 1B). The low acceptance rate reflects donor exclusion criteria (**Table S1**, **SDC**, https://links.lww.com/TXD/A846).

### Geographical Distribution and Donor Cause of Death

**FIGURE 2.**
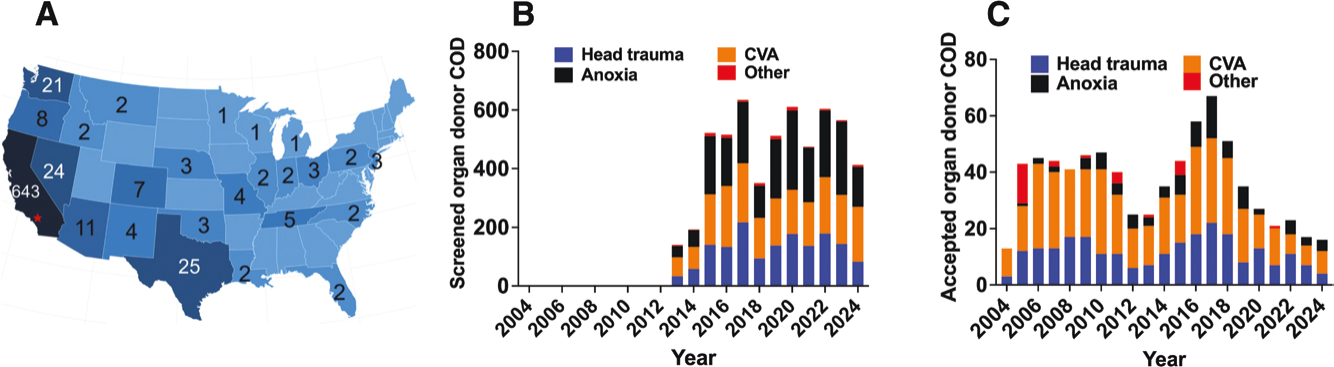
Geographical distribution and donor cause of death of organs offered from 2004 to 2024. A, Geographical distribution of organs accepted for islet isolation. The red star points out the approximate location in Southern California of City of Hope. B, Number of screened donors that had a documented cause of death. Cause of death is noted by different colors. The “other category” designates offers that were not assignable to the categories of head trauma, CVA, or anoxia. C, Number of accepted pancreata from donors that had a documented cause of death. CVA, cardiovascular disease.

Over 80% of accepted organs came from within 1200 miles of City of Hope (Figure 2A). Most organs used for research, 696 (88.9%), originated in California, Nevada, Washington, and Oregon. The causes of death among donors were head trauma, cardiovascular accident, and anoxia (Figure 2B and C). Head trauma and CVA were more prevalent among accepted organs (Figure 2C).

### Donation Death Classification Does not Impact the Quality of Isolated Islets

Historically, islets from DCD donors have been used infrequently for clinical IT.^27-29^ To test our hypothesis, we compared outcomes and quality between islet preparations from DCD and DBD donors (Table 1). The comparison of DCD to DBD islets used pancreata processed for research only, regardless of the donor HbA1c levels. An islet yield of 250 000 islet equivalent (IEQ) was used to identify success of isolation, as implemented by others.^30,31^ This cutoff was used only for confirming whether the islet isolation itself was successful without reference to a decision to use the islets for transplantation. The islet yield of 300 000 IEQ was used as an indicator of transplantable islet yield for several reasons. First, for a qualified transplantation, islet yield should be >5000 IEQ/kg of the recipient’s body weight. Indeed, up to 60 kg recipient body weight was assumed to ensure sufficient IEQ.^32^ These guidelines imply that the total islet yield should be >300 000 IEQ. Second, the results herein derived from transplant cohorts are consistent with the islet yield of 300 000 IEQ. Hence, a 300 000 IEQ islet yield was used to identify the transplantable islets. Within these parameters, no significant differences were found between the DBD and DCD groups in donor age, BMI, cold ischemia time, digestion switch time, islet yield (success >250 000 IEQ), purity (high >80%), recovery (high >75%), post-culture viability, transplantable islet preparations, and insulin stimulation index. DCD organs experienced a significant median warm ischemia interval of 22 min. Interestingly, differences in blood glucose levels from diabetic mice after transplantation using islets from either DCD or DBD donors not only failed to reach significance but trended better in animals given DCD islets (Table 1). Going further, results of noninferiority testing between DCD and DBD islets found both groups to be equivalent, both in terms of islet yield (*P* = 0.01, 0.06 for post-culture and post-isolation, respectively) and in the extent to which these islets reversed diabetes in streptozotocin-treated mice (*P* < 0.01; Table 2). It should be pointed out that the noninferiority test was performed only on the donors that had HbA1c levels <6.5%. The donors with elevated HbA1c levels (≥6.5%) showed significant impaired functionality and were not include in the analysis. A significant difference was observed between the groups in the days of hospitalization, with the DCD group having a median of 5.0 d compared with 4.0 d in the DBD group.

**TABLE 1.** Impact of donation classification (DCD versus DBD) on islet outcome and quality

Factors	Overall (n = 589)	DBD (n = 532)	DCD (n = 57)	*P* ^*a*^
Donor characteristics				
Donor age (y)	47 (35–54)	47 (35–54)	47 (39–57)	0.4
Donor BMI (kg/m^2^)	29.6 (25.9–33.6)	29.5 (25.9–33.3)	31.6 (26.8–36.0)	0.093
Days in hospital	4.0 (3.0–6.0)	4.0 (3.0–6.0)	5.0 (4.0–7.0)	<0.001
Cold ischemia time (min)	421 (343–552)	422 (341–564)	392 (350–455)	0.077
Warm ischemia time (min)	0 (0–21)	0 (0–0)	22 (16–25)	<0.001
Pancreas weight (g)	96 (81–112)	95 (81–111)	101 (82–120)	0.2
Islet isolation outcomes				
Digestion switch time (min)	12.0 (11.0–14.0)	12.0 (11.0–14.0)	12.6 (11.0–13.7)	0.5
Islet yield outcome (success if islet IEQ >250 000)				0.5
Success (%)	214 (37%)	196 (37%)	18 (33%)	
Nonsuccess (%)	365 (63%)	328 (63%)	37 (67%)	
Islet purity (high if purity >80%)				>0.9
High purity (%)	273 (49%)	247 (49%)	26 (50%)	
Low purity (%)	279 (51%)	253 (51%)	26 (50%)	
Islet recovery post-culture (high if recovery >75%)				0.8
High recovery (%)	299 (53%)	271 (53%)	28 (52%)	
Low recovery (%)	263 (47%)	237 (47%)	26 (48%)	
Islet quality control parameters				
Islet viability post-culture (%)	96.0 (93.9–97.8)	96.0 (93.9–97.7)	95.5 (93.9–98.0)	>0.9
Transplantable islet preparations (defined as islet yield ≥300 000 IEQ and viability post-culture ≥93%)^*b*^				0.5
Transplantable (%)	111 (19%)	102 (19%)	9 (16%)	
Suboptimal (%)	478 (81%)	430 (81%)	48 (84%)	
Islet perifusion stimulation index	5.3 (3.8–8.1)	5.4 (3.8–8.2)	4.5 (2.8–5.4)	0.3
Percent reversal of diabetes in mice	67 (0–100)	67 (0–100)	75 (0–100)	0.7

Data are expressed as median (Q1–Q3; Q1 = 25th percentile, Q3 = 75th percentile) or n (%).

^*a*^Wilcoxon rank-sum test; Pearson chi-square test.

^*b*^The criteria were selected according to the results derived from transplant cohorts in this study (average islet yield >300 000 IEQ and viability 93%).

BMI, body mass index; DBD, donation after brain death; DCD, donation after circulatory death; IEQ, islet equivalent.

**TABLE 2.** Impact of donation classification (DCD versus DBD) on islet outcome and quality

Characteristics	DBD	DCD	Difference (95% CI)^*a*^	*P* ^*a*^	Equivalence analysis	
					Bounds	*P*
Islet yield post-isolation (IEQ)	N = 420	N = 45	20 633 (–17 603 to 58 868)	0.3	48 761	0.06
	211 042 (136 200–299 308)	167 617 (107 217–296 583)				
Islet yield post-culture (IEQ)	N = 396	N = 45	3556 (–25 063 to 32 176)	0.8	34 127	0.01
	147 509 (91 512–209 892)	135 133 (81 983–214 000)				
Percent reversal of diabetes in mice (%)	N = 246	N = 30	0.44 (–17 to 18)	>0.9	30	<0.01
	75 (17–100)	88 (0–100)				

Data are expressed as median (Q1–Q3; Q1 = 25th percentile, Q3 = 75th percentile).

^*a*^Welch 2-sample *t* test.

CI, confidence interval; DBD, donation after brain death; DCD, donation after circulatory death; IEQ, islet equivalent.

### Research- and Clinical-grade Islet Donor Metrics Vary

**FIGURE 3.**
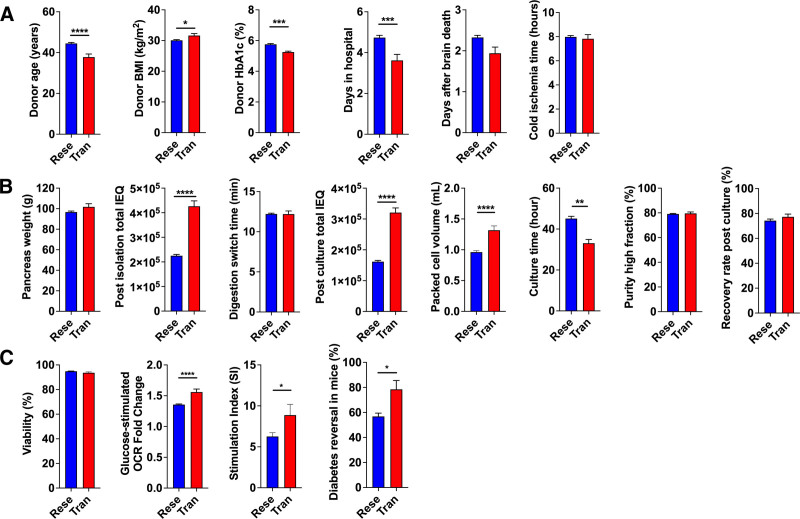
Comparisons of islet isolations between research (Rese) and transplant (Tran) cohorts accepted from 2004 to 2024. A, Donor demographics. Bar graphs show the average donor age (n = 780), BMI (n = 775), HbA1c (n = 705), days in the hospital (n = 739), days after brain death (n = 639), and cold ischemia time (n = 755). B, Islet isolation outcomes. Bar graphs show the average pancreas weight (n = 765), post-isolation total IEQ (n = 754), digestion switch time (n = 764), post-culture total IEQ (n = 733), packed tissue volume (n = 740), culture time (n = 692), purity high fraction (n = 743), and recovery rate post-isolation (n = 744). C, Bar graphs show the average viability (n = 745), the glucose-stimulated OCR fold change (n = 301), the SI as derived from the results of the glucose-stimulated insulin secretion assay (n = 745), and the rate of normalization of blood glucose levels in diabetic mice after islet transplantation (n = 333). Nonparametric 2-tailed *t* tests were used to compare the research and transplant cohorts. The data are expressed as mean ± SEM. *P < 0.05; **P < 0.01; ***P < 0.001; and ****P < 0.0001. BMI, body mass index; HbA1c, hemoglobin A1c; IEQ, islet equivalent; OCR, oxygen consumption rate; SI, stimulation index.

Enlarging on this, comparison was made between research- and clinical-grade islet preparations starting with the organ donor demographic and biometric data. Donor age averaged 44 ± 0.5 y (range, 14–72 y; Figure 3A). This is similar to reported mean donor ages (45 ± 4.7 y).^33^ Donors were younger among islet preparations isolated for clinical IT (Tran, 37.8 ± 1.5; Rese, 44.5 ± 0.5; *P* < 0.0001; Figure 3A). Average donor BMI (30.2 ± 0.2 kg/m^2^) was within the obese range (Figure 3A) and was greater in donors where islets went to clinical IT (Tran, 31.6 ± 0.6; Rese, 30.1 ± 0.2; *P* < 0.05; Figure 3A), perhaps because the organs were larger^34^ and could provide more IEQ.^30^ However, in our experience, larger islets were less functional.^35^ Hemoglobin A1c (HbA1c) averaged 5.7% ± 0%, although some donors were prediabetic (5.7%–6.5%)^36^ or diabetic (≥6.5%; Figure 3A). HbA1c was lower and less variable in donors where islets went to clinical IT (Tran, 5.3 ± 0.1; Rese, 5.8 ± 0.1; *P* < 0.001; Figure 3A). Donor days in the hospital averaged 4.6 ± 0.1 and increased over the length of the study (Figure 3A). Total hospital days were less in donors where islets went to clinical IT (Tran, 3.6 ± 0.3; Rese, 4.7 ± 0.1; *P* < 0.001; Figure 3A). Donor days after brain death averaged 2.3 ± 0 d (Figure 3A) and trended upward with time (not shown). This might have been the result of an expansion in the organ acceptance criteria.^37^ The cold ischemia time of the organs averaged 8.0 ± 0.1 h (Figure 3A). The donor days after brain death and cold ischemia time were not significantly different between islets used in research and islets demarcated for transplantation (Figure 3A).

### Clinical-grade Islets are Superior in Several Isolation Metrics

Donor pancreas weight averaged 97.2 ± 1.0 grams (range, 24.5–205.9 grams; Figure 3B). Digestion switch time, defined as the time that the pancreas tissue fragments are exposed to enzyme digestion, averaged 12.2 ± 0.1 min (range, 4.0–29.0 min; Figure 3B). The length of enzymatic digestion of the pancreas is influenced by the experience of the isolation team, the extracellular matrix content of the organ, and the activity of the digestion enzyme.^38,39^ Islet culture time averaged 44.0 ± 1.9 h (range, 1.7–163.5; Figure 3B) and was less among islets used for clinical IT (Tran, 33.0 ± 1.8; Rese, 45.1 ± 1.1; *P* < 0.01). Packed tissue volume, the volume of islets from the high purity (>70%) fraction, averaged 1.0 ± 0 mL (range, 0.1–9.0 mL; Figure 3B). This measurement is another marker of islet isolation success.^27,40^ Here too, the packed tissue volume was larger among islets used in clinical IT (Tran, 1.3 ± 0.1; Rese, 1.0 ± 0; *P* < 0.0001; Figure 3B). The islet isolation process was deemed successful based on the average purity of the high fraction islets (79.2% ± 0.3%; range, 15.0–95.0; Figure 3B), and this was the same regardless of the islet usage.

### Clinical-grade Islets Have Better Functionality

Post-culture viability of islets averaged 94.8% (63.8%–100; Figure 3C). The islet OCR was 1.4 (range, 1.0–2.1), consistent with high metabolic function in the islet preparations (Figure 3C). The islets used in clinical IT exhibited significantly higher OCR fold change compared with the islets used for research (Tran, 1.6 ± 0.2; Rese, 1.4 ± 0.2; *P* < 0.0001; Figure 3C). The OCR fold change data from 2004 to 2014 was not available. The stimulated insulin release index, defined as the maximum glucose-stimulated insulin release over basal release, averaged 6.6 (range, 0.9–81.8; Figure 3C) and was greater in transplant versus research islets (Figure 3C). In diabetic mice, correction of hyperglycemia after IT averaged 57% (range, 0–100%; Figure 3C). Islets used for clinical IT were more effective at correcting blood glucose levels in diabetic mice than islets used for research (Tran, 78.5 ± 34.7; Rese, 57.0 ± 43.8; *P* < 0.05; Figure 3C).

### Transplant and Research Islet Isolation Quality Control Assay Results

Overall, the isolation process yielded quality islets for clinical IT and research. When data were segregated by final islet disposition, islets that were used in clinical IT were found to be of higher quality compared with islets used in research (Figure 3C).

### Donor HbA1c Levels and Islet Outcome and Quality

We compared islet outcome and quality within ranges of HbA1c: nondiabetic (HbA1c <5.7%; n = 425); prediabetic (5.7 ≤HbA1c <6.5%; n = 160); and diabetic (HbA1c ≥6.5%; n = 74; **Table S2**, **SDC**, https://links.lww.com/TXD/A846). Significant differences (*P* < 0.05) were observed in donor age (median of 44, 50, and 52 y; *P* < 0.001), donor BMI (median of 29.1, 29.7, and 31.8 kg/m^2^; *P* = 0.005), days in hospital (median of 4.0, 4.0, and 4.0 d; *P* = 0.039), and digestive switch time (median of 12.0, 11.7, and 13.0 min; *P* < 0.001) in the nondiabetic, prediabetic, and diabetic groups. Furthermore, yield outcome (success >250 000 IEQ)^30,31^ was significantly different (*P* = 0.003), with success rates of 39%, 36%, and 18% in the nondiabetic, prediabetic, and diabetic groups. A significant difference (*P* = 0.022) was found in achieving transplantable islet preparations, being 18% and 17% in the nondiabetic and prediabetic groups compared with 5% in the diabetic group. Median islet perifusion insulin levels varied significantly among the nondiabetic, prediabetic, and diabetic groups being 6.0, 4.0, and 3.0 (*P* = 0.006). Furthermore, islet isolations from donors with an HbA1c ≥6.5% did not correct blood glucose levels when transplanted into diabetic mice, whereas 75% of the islets from nondiabetic donors restored blood glucose levels to within normal ranges. No significant differences were found in cold ischemia time, pancreas weight, islet purity, recovery, viability post-culture, and islet perifusion stimulation index (**Table S2**, **SDC**, https://links.lww.com/TXD/A846).

## DISCUSSION

Islet isolation data from other centers >30 y is summarized in **Table S3** (**SDC**, https://links.lww.com/TXD/A846). In some instances, demographic data were limited. Raw unadjusted data were uncommon. This may be secondary to the evolving nature of the technology and the increasing interest in and use of isolated human islets. The present study gathered data from all human islet isolations performed >20 y at an active clinical IT center. In this series, a minority of offered organs (<10%) were accepted for islet isolation. Of these, 1 quarter of the islet preparations for use in clinical IT ultimately did not qualify. This was attributable to quality assay results and there being >1 individual at a time on the transplantation list. Nonetheless, 69 islet preparations were transplanted into 36 individuals with severe T1D.

In 2023, IT was recognized by the Food and Drug Administration as a cellular therapy for the treatment of individuals with T1D.^41^ Transplantation DCD donor pancreata could be a valuable source of isolated islets for clinical transplantation that would help address the scarcity and demand for the cell therapy.^42^ In spite of this, the status of DCD pancreata remains indeterminate because of quality concerns and the impact of warm ischemia engendered by the donation process.^43,44^ The intent of this analysis was to compare the quality of islets from DCD and DBD pancreata. Supporting our hypothesis, equivalency testing found that DCD islets were not inferior to DBD islets in regard to islet yield and reversal of diabetes after transplantation into mice. Additional analysis of multiple islet quality metrics demonstrated that DCD islets were equal to standard-of-care DBD islets. Among a portfolio of parameters studied, no statistical differences between the 2 groups were found. Although islet yields from DCD pancreases were lower immediately post-isolation compared with DBD organs,^43^ in individuals given DCD islets clinical outcomes such as function at 1 and 2 y were similar to those given DBD islets.^45^ There are strategies to mitigate the risks associated with islets from DCD organs. For instance, limiting organ warm ischemia time to ≤10 min to preserve islet yield,^43^ and the use of Institute Georges Lopez-1 cold preservation solution.^43^ Another novel approach is abdominal normothermic regional perfusion of pancreata in DCD individuals. The technique improved islet yield without loss of function compared with standard DCD and DBD islets.^46^

As far is known, this is among the first applications of equivalence testing to isolated islet quality metrics. The current study extends prior work^45,47,48^ and provides a rationale for using DCD islets for clinical IT. Also supportive is data showing that solid organs from DCD donors such as the kidney, liver, lungs, pancreas, and heart were therapeutic.^49-52^

Another finding is that islet isolation under a current good manufacturing practice protocol yielded large numbers of quality islets, a result that remained stable >2 decades. For example, islet yield averaged 243 341 IEQ (range, 8433–1 186 678), islet purity 79.2% ± 0.3 % (range, 15.0–95.0), and islet viability 94.8% (range, 63.8–100). Related to this, we considered that donor biometrics would negatively impact the quality of isolated human islets as reported by others.^17^ Among these, donor age, BMI, HbA1c, and duration of hospital stay impacted islet isolation outcomes and separated islets prepared for clinical IT from those designated for research.

Transplantation of human islets into diabetic mice is not a required step to qualify islets for clinical transplantation.^53^ However, this bioassay provides useful information relevant to islet engraftment. Furthermore, the data obtained allows for correlative analysis of donor HbA1c levels and murine outcomes. OCR, another islet quality control mainstay, assesses islet response to redox stress^54,55^ and, with islet size, predicted islet graft function.^23,56^ This is germane, as we found that isolated islets secrete large amounts of thrombospondin-1,^57^ an anti-angiogenic protein upregulated by hypoxia inducible factor^58^ that limits progrowth vascular endothelial growth factor receptor^59^ and vascular nitric oxide^60^ to impede angiogenesis, blood flow, and solid organ transplantation.^61^ Furthermore, thrombospondin-1 signaling was inferred to limit experimental IT.^62^

This study demonstrated a significant association between elevated donor HbA1c levels and inferior islet metrics. Donors with HbA1c levels ≥6.5% were significantly older and had higher BMI compared with the nondiabetic and prediabetic donor groups. These characteristics were associated with morphologic changes in the pancreata including increased adipose content and tissue fibrosis.^63^ Related to this, we found that the organs from diabetic donors required longer digestion times. To this point, the success rate of islet yields (>250 000 IEQs) declined markedly from 39% in organs from nondiabetic individuals to 12% in organs from the diabetic group. Even more, the proportion of preparations suitable for clinical IT dropped to 5% in the diabetic cohort. These data align with other reports^19,64^ and highlight the functional impact of donor metabolic status on the quality of isolated islets and emphasize the value of HbA1c in donor evaluation.

Several caveats arose with the present study. The information base was accumulated >20 y and, in some instances as noted, was incomplete. This made retrospective interpretation a challenge. Also, changes in the cold preservation solution^65^ and digestion enzyme^39^ might have affected islet isolation metrics. Other factors inherent to the isolation process, such as trimming the organ before digestion, the speed with which the organ is processed, and variation in manual shaking of the digestion chamber, could have a meaningful effect on the quantity and quality of the islets. These sorts of factors are difficult to measure and to track. The precise effects upon islet isolation success of the above-mentioned confounders, in combination with donor disease state, remain unknown.

In conclusion, the results from analysis of 801 consecutive islet isolation >2 decades from a single center found that DCD islets were equivalent to standard-of-care DBD islets. Expanded acceptance of organs from DCD donors should be given serious consideration.

## ACKNOWLEDGMENTS

The authors gratefully acknowledge the organ donors and their families. The authors thank Dr Yoko Mullen for establishing the Southern California Islet Cell Resource Center.

## Supplementary Material



## References

[1] Brennan DC Kopetskie HA Sayre PH. Long-term follow-up of the Edmonton protocol of islet transplantation in the United States. Am J Transplant. 2016;16:509–517. 26433206 10.1111/ajt.13458

[2] Hering BJ Clarke WR Bridges ND; Clinical Islet Transplantation Consortium. Phase 3 trial of transplantation of human islets in type 1 diabetes complicated by severe hypoglycemia. Diabetes Care. 2016;39:1230–1240. 27208344 10.2337/dc15-1988 PMC5317236

[3] Foster ED Bridges ND Feurer ID; Clinical Islet Transplantation Consortium. Improved health-related quality of life in a phase 3 islet transplantation trial in type 1 diabetes complicated by severe hypoglycemia. Diabetes Care. 2018;41:1001–1008. 29563196 10.2337/dc17-1779 PMC5911786

[4] Berney T Andres A Bellin MD; International Islet Transplant Centers. A worldwide survey of activities and practices in clinical islet of Langerhans transplantation. Transpl Int. 2022;35:10507. 36033644 10.3389/ti.2022.10507 PMC9402897

[5] Marfil-Garza BA Shapiro AMJ Kin T. Clinical islet transplantation: current progress and new frontiers. J Hepatob Pancreat Sci. 2021;28:243–254. 10.1002/jhbp.891 33417749

[6] Qi M Kinzer K Danielson KK. Five-year follow-up of patients with type 1 diabetes transplanted with allogeneic islets: the UIC experience. Acta Diabetol. 2014;51:833–843. 25034311 10.1007/s00592-014-0627-6 PMC4801517

[7] Rother KI Harlan DM. Challenges facing islet transplantation for the treatment of type 1 diabetes mellitus. J Clin Invest. 2004;114:877–883. 15467822 10.1172/JCI23235 PMC518676

[8] Wong JM Pepper AR. Status of islet transplantation and innovations to sustainable outcomes: novel sites, cell sources, and drug delivery strategies. Front Transplant. 2024;3:1485444. 39553396 10.3389/frtra.2024.1485444 PMC11565603

[9] Suntharalingam C Sharples L Dudley C. Time to cardiac death after withdrawal of life-sustaining treatment in potential organ donors. Am J Transplant. 2009;9:2157–2165. 19681825 10.1111/j.1600-6143.2009.02758.x

[10] Anazawa T Saito T Goto M. Long-term outcomes of clinical transplantation of pancreatic islets with uncontrolled donors after cardiac death: a multicenter experience in Japan. Transplant Proc. 2014;46:1980–1984. 25131088 10.1016/j.transproceed.2014.06.006

[11] Walker S Appari M Forbes S. Considerations and challenges of islet transplantation and future therapies on the horizon. Am J Physiol Endocrinol Metab. 2022;322:E109–E117. 34927459 10.1152/ajpendo.00310.2021

[12] Qi M Luis V Bilbao S. Sodium levels of human pancreatic donors are a critical factor for determination of islet efficacy and survival. Am J Physiol Endocrinol Metab. 2015;308:E362–E369. 25537495 10.1152/ajpendo.00443.2014

[13] Ricordi C Lacy PE Scharp DW. Automated islet isolation from human pancreas. Diabetes. 1989;38(Suppl 1):140–142. 2642838 10.2337/diab.38.1.s140

[14] Kauffman HM McBride MA Delmonico FL. First report of the United Network for Organ Sharing Transplant Tumor Registry: donors with a history of cancer. Transplantation. 2000;70:1747–1751. 11152107 10.1097/00007890-200012270-00014

[15] Brissova M Niland JC Cravens J. The Integrated Islet Distribution Program answers the call for improved human islet phenotyping and reporting of human islet characteristics in research articles. Diabetologia. 2019;62:1312–1314. 31089753 10.1007/s00125-019-4876-3 PMC7365209

[16] Takita M Matsumoto S Noguchi H. One hundred human pancreatic islet isolations at Baylor Research Institute. Proc. 2010;23:341–348. 10.1080/08998280.2010.11928648 PMC2943445 20944753

[17] Kaddis JS Danobeitia JS Niland JC. Multicenter analysis of novel and established variables associated with successful human islet isolation outcomes. Am J Transplant. 2010;10:646–656. 20055802 10.1111/j.1600-6143.2009.02962.x PMC2860018

[18] Shapiro AM. State of the art of clinical islet transplantation and novel protocols of immunosuppression. Curr Diab Rep. 2011;11:345–354. 21830042 10.1007/s11892-011-0217-8

[19] Qi M McFadden B Valiente L. Human pancreatic islets isolated from donors with elevated HbA1c levels: islet yield and graft efficacy. Cell Transplant. 2015;24:1879–1886. 25198342 10.3727/096368914X683548

[20] Khiatah B Qi M Wu Y. Pancreatic human islets and insulin-producing cells derived from embryonic stem cells are rapidly identified by a newly developed Dithizone. Sci Rep. 2019;9:9295. 31243300 10.1038/s41598-019-45678-y PMC6594947

[21] Qi M Kaddis JS Chen KT. Chronic marijuana usage by human pancreas donors is associated with impaired islet function. PLoS One. 2021;16:e0258434. 34705837 10.1371/journal.pone.0258434 PMC8550598

[22] Omori K Qi M Salgado M. A scalable human islet 3D-culture platform maintains cell mass and function long-term for transplantation. Am J Transplant. 2024;24:177–189. 37813189 10.1016/j.ajt.2023.10.001

[23] Komatsu H Qi M Gonzalez N. A multiparametric assessment of human islets predicts transplant outcomes in diabetic mice. Cell Transplant. 2021;30:9636897211052291. 34628956 10.1177/09636897211052291 PMC8504220

[24] Gaber AO Fraga D Kotb M. Human islet graft function in NOD-SCID mice predicts clinical response in islet transplant recipients. Transplant Proc. 2004;36:1108–1110. 15194386 10.1016/j.transproceed.2004.04.055

[25] Caiazzo R Gmyr V Kremer B. Quantitative in vivo islet potency assay in normoglycemic nude mice correlates with primary graft function after clinical transplantation. Transplantation. 2008;86:360–363. 18645503 10.1097/TP.0b013e31817ef846

[26] Friberg AS Brandhorst H Buchwald P. Quantification of the islet product: presentation of a standardized current good manufacturing practices compliant system with minimal variability. Transplantation. 2011;91:677–683. 21248660 10.1097/TP.0b013e31820ae48e

[27] Ricordi C Goldstein JS Balamurugan AN. National Institutes of Health-Sponsored Clinical Islet Transplantation Consortium Phase 3 Trial: manufacture of a complex cellular product at eight processing facilities. Diabetes. 2016;65:3418–3428. 27465220 10.2337/db16-0234 PMC5079635

[28] Kandeel F El-Shahawy M Singh G. Towards a rational balanced pancreatic and islet allocation schema. Cell Transplant. 2021;30:9636897211057130. 34757859 10.1177/09636897211057130 PMC8586185

[29] Proneth A Schnitzbauer AA Zeman F. Extended pancreas donor program—the EXPAND study rationale and study protocol. Transplant Res. 2013;2:12. 23816330 10.1186/2047-1440-2-12 PMC3716891

[30] Nano R Clissi B Melzi R. Islet isolation for allotransplantation: variables associated with successful islet yield and graft function. Diabetologia. 2005;48:906–912. 15830183 10.1007/s00125-005-1725-3

[31] Hanley SC Paraskevas S Rosenberg L. Donor and isolation variables predicting human islet isolation success. Transplantation. 2008;85:950–955. 18408573 10.1097/TP.0b013e3181683df5

[32] Matsumoto S Noguchi H Hatanaka N. Estimation of donor usability for islet transplantation in the United States with the kyoto islet isolation method. Cell Transplant. 2009;18:549–556. 19775516 10.1177/096368970901805-610

[33] Balamurugan AN Loganathan G Bellin MD. A new enzyme mixture to increase the yield and transplant rate of autologous and allogeneic human islet products. Transplantation. 2012;93:693–702. 22318245 10.1097/TP.0b013e318247281b PMC3314155

[34] Brandhorst H Brandhorst D Hering BJ. Body mass index of pancreatic donors: a decisive factor for human islet isolation. Exp Clin Endocrinol Diabetes. 1995;103(Suppl 2):23–26. 8839248 10.1055/s-0029-1211388

[35] Komatsu H Salgado M Gonzalez N. High fractions of large islets in human islet preparations detrimentally affect posttransplant outcomes in streptozotocin-induced diabetic immunodeficient mice. Pancreas. 2020;49:650–654. 32433402 10.1097/MPA.0000000000001541

[36] American Diabetes A. Diagnosis and classification of diabetes mellitus. Diabetes Care. 2011;34(Suppl 1):S62–S69. 21193628 10.2337/dc11-S062 PMC3006051

[37] Kulkarni RN Stewart AF. Summary of the Keystone islet workshop (April 2014): the increasing demand for human islet availability in diabetes research. Diabetes. 2014;63:3979–3981. 25414011 10.2337/db14-1303 PMC4238004

[38] Khiatah B Tucker A Chen KT. Evaluation of collagenase gold plus BP protease in isolating islets from human pancreata. Islets. 2018;10:51–59. 29381419 10.1080/19382014.2017.1417716 PMC5895173

[39] Qi M Valiente L McFadden B. The choice of enzyme for human pancreas digestion is a critical factor for increasing the success of islet isolation. Transplant Direct. 2015;1:e14. 26146662 10.1097/TXD.0000000000000522 PMC4486320

[40] Pisania A Weir GC O’Neil JJ. Quantitative analysis of cell composition and purity of human pancreatic islet preparations. Lab Invest. 2010;90:1661–1675. 20697378 10.1038/labinvest.2010.124 PMC2966538

[41] Wang Y Chen Y McGarrigle J. Cell therapy for T1D beyond BLA: gearing up toward clinical practice. Diabetes Ther. 2025;16:1125–1138. 40214896 10.1007/s13300-025-01732-9 PMC12085407

[42] Berney T Boffa C Augustine T. Utilization of organs from donors after circulatory death for vascularized pancreas and islet of Langerhans transplantation: recommendations from an expert group. Transpl Int. 2016;29:798–806. 26340064 10.1111/tri.12681

[43] De Paep DL Van Hulle F Ling Z. Lower beta cell yield from donor pancreases after controlled circulatory death prevented by shortening acirculatory warm ischemia time and by using IGL-1 cold preservation solution. PLoS One. 2021;16:e0251055. 33939760 10.1371/journal.pone.0251055 PMC8092795

[44] De Paep DL Jacobs-Tulleneers-Thevissen D. Warm ischemia time influences human islet cell isolation yield when assessed as beta cell number but not as islet equivalent number. Am J Transplant. 2021;21:3814–3815. 34008319 10.1111/ajt.16691

[45] Doppenberg JB Nijhoff MF Engelse MA. Clinical use of donation after circulatory death pancreas for islet transplantation. Am J Transplant. 2021;21:3077–3087. 33565712 10.1111/ajt.16533 PMC8518956

[46] Doppenberg JB van Rooden RM van Dijk MC. Abdominal normothermic regional perfusion after donation after circulatory death improves pancreatic islet isolation yield. Am J Transplant. 2025;25:594–601. 39366509 10.1016/j.ajt.2024.09.034

[47] Parente A Verhoeff K Kin T. Evaluating islet cell isolation and transplantation from donors following medical assistance in dying. Transplant Direct. 2024;10:e1667. 38911274 10.1097/TXD.0000000000001667 PMC11191926

[48] De Paep DL Van Hulle F Ling Z. Utility of islet cell preparations from donor pancreases after euthanasia. Cell Transplant. 2022;31:9636897221096160. 35583214 10.1177/09636897221096160 PMC9125111

[49] van Rijn R Endo C Kucukerbil EH. Long-term Follow-up after hypothermic oxygenated machine perfusion in DCD liver transplantation: results of a randomized controlled multicenter trial (DHOPE-DCD). Ann Surg. 2025;282:717–724. 41082480 10.1097/SLA.0000000000006876 PMC12513034

[50] Oki R Rocha I Al-Juburi S. The individual impact of machine perfusion on liver and kidney on donor expansion in simultaneous liver and kidney transplantation. Transpl Int. 2025;38:14807. 40994627 10.3389/ti.2025.14807 PMC12454159

[51] Duehmke R Hassan M Page A. Impact of donation after circulatory death heart transplantation on clinical outcomes after listing for heart transplantation. Eur J Cardiothorac Surg. 2025;67:ezaf315. 40985735 10.1093/ejcts/ezaf315

[52] Bleszynski MS Parmentier C Torres-Hernandez A. Pancreas transplantation with grafts obtained from donation after cardiac death or donation after brain death results in comparable outcomes. Front Transplant. 2023;2:1176398. 38993888 10.3389/frtra.2023.1176398 PMC11235253

[53] Glieberman AL Pope BD Melton DA. Building biomimetic potency tests for islet transplantation. Diabetes. 2021;70:347–363. 33472944 10.2337/db20-0297 PMC7881865

[54] Komatsu H Kandeel F Mullen Y. Impact of oxygen on pancreatic islet survival. Pancreas. 2018;47:533–543. 29621044 10.1097/MPA.0000000000001050 PMC5943071

[55] Kato H Salgado M Mendez D. Biological hypoxia in pre-transplant human pancreatic islets induces transplant failure in diabetic mice. Sci Rep. 2024;14:12402. 38811610 10.1038/s41598-024-61604-3 PMC11137081

[56] Pepper AR Hasilo CP Melling CW. The islet size to oxygen consumption ratio reliably predicts reversal of diabetes posttransplant. Cell Transplant. 2012;21:2797–2804. 22943589 10.3727/096368912X653273

[57] Erdem N Chen KT Qi M. Thrombospondin-1, CD47, and SIRPalpha display cell-specific molecular signatures in human islets and pancreata. Am J Physiol Endocrinol Metab. 2023;324:E347–E357. 36791324 10.1152/ajpendo.00221.2022 PMC11967708

[58] Labrousse-Arias D Castillo-Gonzalez R Rogers NM. HIF-2alpha-mediated induction of pulmonary thrombospondin-1 contributes to hypoxia-driven vascular remodelling and vasoconstriction. Cardiovasc Res. 2016;109:115–130. 26503986 10.1093/cvr/cvv243 PMC4692290

[59] Kaur S Martin-Manso G Pendrak ML. Thrombospondin-1 inhibits VEGF receptor-2 signaling by disrupting its association with CD47. J Biol Chem. 2010;285:38923–38932. 20923780 10.1074/jbc.M110.172304 PMC2998110

[60] Isenberg JS Ridnour LA Dimitry J. CD47 is necessary for inhibition of nitric oxide-stimulated vascular cell responses by thrombospondin-1. J Biol Chem. 2006;281:26069–26080. 16835222 10.1074/jbc.M605040200

[61] Rogers NM Zhang ZJ Wang JJ. CD47 regulates renal tubular epithelial cell self-renewal and proliferation following renal ischemia reperfusion. Kidney Int. 2016;90:334–347. 27259369 10.1016/j.kint.2016.03.034

[62] Ghimire K Kale A Li J. A metabolic role for CD47 in pancreatic beta cell insulin secretion and islet transplant outcomes. Sci Transl Med. 2023;15:eadd2387. 37820008 10.1126/scitranslmed.add2387

[63] Wang CH Orr C Hacker-Stratton J. Shorter digestion times of donor islets is associated with better islet graft function after islet transplantation. Cell Transplant. 2025;34:9636897241310989. 39881535 10.1177/09636897241310989 PMC11780635

[64] Wang LJ Kin T O’Gorman D. A multicenter study: North American islet donor score in donor pancreas selection for human islet isolation for transplantation. Cell Transplant. 2016;25:1515–1523. 26922947 10.3727/096368916X691141 PMC5167495

[65] Paushter DH Qi M Danielson KK. Histidine-tryptophan-ketoglutarate and University of Wisconsin solution demonstrate equal effectiveness in the preservation of human pancreata intended for islet isolation: a large-scale, single-center experience. Cell Transplant. 2013;22:1113–1121. 23031661 10.3727/096368912X657332 PMC3759240

