# Supplementary material for: Donation After Circulatory Death Islets are Comparable to Standard-of-Care Donation After Brain Death Islets: Analysis of 801 Consecutive Human Islet Isolations
Source: Transplant Direct. 2026 Mar 20;12(4):e1930. doi: 10.1097/TXD.0000000000001930 (PMC13008207; doi:10.1097/TXD.0000000000001930)
Supplement: Supplementary file 1 [file txd-12-e1930-s001.pdf]

Donation After Circulatory Death Islets Are Comparable to Standard-of-Care Donation After Brain Death Islets: Analysis of 801 Consecutive Human Islet Isolations

Supplemental Digital Content

Donor screening

The donor screening for all organs was conducted by the organ acceptance team. The screening process began with the identification of potential donors through organ procurement organizations. Donor eligibility was determined based on age, body mass index, cause of death, and medical history. Blood samples were tested for infectious diseases. The pancreas was visualized by the procurement surgeon to exclude anatomical abnormalities. Acceptance and decline of acceptance were documented. The islet isolation team was informed when an organ was accepted for processing. The selection criteria for pancreas donors for transplant and research are listed in **Table S1.**

Human islet isolation

Human islet isolation was performed under a protocol approved by the City of Hope Institutional Review Board (IRB 01046), adhering to current Good Manufacturing Practice (cGMP) standards. Donor documentation, including consent forms and medical history, were verified and the pancreas was processed in a biological safety cabinet. To test for microbial contamination, organ cold preservation solution was sampled. Antibiotics were added to the trimming solution employed during organ dissection. The surrounding adipose and connective tissue was removed, and the pancreas was weighed to calculate the amount of enzyme required for organ digestion. The pancreas was transected at the neck of the organ and cannulated via the main pancreatic duct using a 16 or 20 gauge angio-catheter (Cardinal Health, Ontario, CA) that was secured with 2-0 silk suture. A tissue biopsy was collected for histological evaluation. Enzyme solution was prepared using either Liberase HI (Roche Diagnostics, Roche Applied Science, Indianapolis, IN), Liberase

MTF C/T (Roche Diagnostics), or Collagenase NB1 with NP (Nordmark Biochemicals, Germany). Enzymatic perfusion was carried out with a customized perfusion system (Biorep Technologies, Miami Lakes, FL) under controlled pressure and flow: first at 60–80 mm Hg for 5 minutes followed by 160–180 mm Hg for 5 minutes, with the temperature <14°C. Following perfusion, the pancreas was cut into 6-10 pieces and placed in a Ricordi Digestion Chamber containing 7–10 marbles and enzyme solution. The chamber was connected to a circulating water bath maintained at 35–38°C. During the digestion process, aliquots were periodically stained with dithizone (DTZ) and examined microscopically to determine the degree of islet release. Digestion was halted with cold culture medium when ~50% of islets were free of the surrounding pancreatic tissue. Islets were purified using a COBE 2991 cell processor. Transplant-grade islets were cultured in CMRL 1066 Supplemented CIT Modification culture media (Corning, Glendale, AZ) supplemented with 0.5% human serum albumin (Baxalta-Takeda, Cambridge, MA) and insulin-like growth factor-1 (0.1 μg/mL) (Repligen, Waltham, MA) under cGMP conditions at 22–37°C with 5% CO_2 for up to 72 hours. Research-grade islets were cultured in PIM-R culture media (Prodo Laboratories Inc., Aliso Viejo, CA) supplemented with PIM (G) (Prodo Laboratories Inc.) and 5% human serum (GeminiBio, West Sacramento, CA) at 27–37°C with 5% CO_2 for up to 5 days.

Islet counting and viability assessment. Islet samples (100 μL/sample, 2-4 samples/batch) were taken at the end of isolation and after culture using a Drummond pipette. The islet equivalents (IEQ), islet particle number (IPN), and islet purity (%) were determined manually by staining islets with dithizone (DTZ or iDTZ) followed by microscopic inspection. The islet viability was determined by staining islets with 0.48 μM fluorescein diacetate and 10 μg/mL propidium iodide solution (Millipore Sigma, St. Louis, MO). Viability (%) was manually assessed under a IXDP50 Olympus microscope with a DP74 camera using a 10x objective lens (Olympus America, Center

Valley, PA).

Perifused glucose-stimulated insulin release assay. Islets (350 IEQ) were placed into a column filled with Cytodex 1 beads (Cat# 17044801, Cytiva, Marlborough, MA) and perfused for one hour with low glucose (3 mM) in Krebs-Ringer buffer (KRB) solution (pH 7.4) at 37º C. This was then followed by perfusion with KRB buffer containing 3 mM glucose for 10 minutes, perifusion with 16.8 mM glucose for 20 minutes, and perifusion with 3 mM glucose for 25 minutes. Fluid samples from the column were taken every minute. The insulin concentration of the samples was determined using an ELISA kit (Cat# 10-1113-10, Mercodia Inc., Winston Salem, NC). The stimulation index was calculated as the ratio of the sum of insulin release during the first 13 minutes of exposure to 16.8 mM glucose divided by the insulin release during 13 minutes of exposure to 3 mM glucose.

Static glucose-stimulated insulin release assay. Islets (140 IEQ) were incubated in RPMI 1640 media supplemented with 10 mM HEPES, 10% fetal bovine serum, and 3 mM glucose for ≥ 4 hours. Islets were then placed on a Millicell 12 mm polycarbonate culture insert (pore size 12 μm) (Cat# PIXP01250, Millipore, Sigma), placed in a 24-well plate, and incubated for 60 minutes with KRB solution containing 2.8 mM glucose. Islets were then placed in a new well with KRB containing 2.8 mM glucose for 60 minutes, followed by 60 minutes in the same volume of KRB solution containing 28 mM glucose. An aliquot of the incubation buffer sample was collected after each incubation interval. Insulin levels were determined using an ELISA kit (Mercodia Inc.). The stimulation index was calculated as the insulin in the media sample during 28 mM glucose stimulation for 60 minutes divided by the insulin level in the media during 2.8 mM glucose.

Glucose-stimulated oxygen consumption rate (OCR). Assessment of islet OCR was done using Seahorse XFe24 Islet Capture FluxPak (Cat# 103518-100, Agilent, Santa Clara, CA) in a Seahorse

XFe24 analyzer. The fold change in the OCR was calculated as the difference in the baseline OCR of islets exposed to 3 mM glucose for 49 minutes minus the OCR after exposure to 20 mM glucose for the same period. The maximum OCR was obtained at the last measurement of glucose stimulation. The OCR fold change was defined as the maximum OCR divided by the basal OCR.

***In vivo* islet assay.** Animal experiments were conducted with the approval of the Institutional Animal Care and Use Committee of City of Hope (IACUC #01020). Animals were maintained under pathogen-free conditions at the Animal Resources Center of the Beckman Research Institute of City of Hope. Diabetic 8- to 12-week-old male nonobese severe combined immunodeficient mice (NOD. Cg-*Prkdc*^{scid} /J; Jackson Laboratory, RRID: IMSR JAX:001303) received human islets. Diabetes was induced in the mice with streptozotocin (50 mg/kg i.p daily., Sigma-Aldrich, St Louis, MO) administered over three consecutive days. Hyperglycemic mice (blood glucose levels >350 mg/dL on two consecutive days) received 1,200 IEQ human islets from a single organ placed under the left kidney capsule. Islets from each donor pancreas were transplanted into at least two mice. Post-transplantation, blood glucose levels were determined 2-3 times per week for 28 days using a glucometer (LifeScan, Inc., Milpitas, CA). Mice that maintained blood glucose levels <200 mg/dL over two consecutive days were classified as diabetes-free. Failure to reverse diabetes was defined as instances where blood glucose levels either never fell below 200 mg/dL or initially fell below 200 mg/dL but subsequently rose above this. To confirm that the reversal of diabetes was mediated by the transplanted islets, the islet-bearing kidney was removed 4 weeks after the transplantation.

Table S1. Eligibility and exclusion criteria for donors and organs employed for transplantation and research

	Criteria for Transplant	Criteria for Research
**Basic donor information**		
Blood type	Must be compatible with recipient blood type	Any
Age (years)	15-65	15-65
Sex/Race	Any	Any
Height	Any	Any
Weight	≤ 400 lbs (181.44 kg)	Any
Body mass index	20-45	20-45
Cardiorespiratory arrest downtime	< 15 min	< 30 min
Hospital stay duration	< 10 days	< 14 days
Donation after cardiac/circulatory death	Not acceptable	Warm ischemia time < 30 min
**Donor medical history**		
Acquired Immunodeficiency Syndrome	Not acceptable	Not acceptable
Damage/Trauma to pancreas	Not acceptable	Not acceptable
Diabetes diagnosis	Not acceptable	Case by case
Endocrine and/or autoimmune diseases other than diabetes	If two of more diseases, consult on-call PI/Designee	If two of more diseases, consult on-call PI/Designee
Current history of cancer except basal and or squamous cell	Not acceptable	Not acceptable
Pancreatitis - acute or chronic	Not acceptable	Not acceptable
Recent unexplained weight loss	Not acceptable	Consult on-call PI/Designee
Renal disease/dialysis	Not acceptable	Not acceptable
Use of human growth hormone	Not acceptable	Not acceptable
Chronic disease (HTN, hyperlipidemia, COPD, etc.)	Consult on-call PI/Designee	Consult on-call PI/Designee
**Donor serology**		
Viral status	All negative	All negative
CMV/EBV IgM	Negative	Negative
**Donor characteristics at death**		
Hemodiluted serology sample	Not acceptable	Not acceptable
Serum creatinine	Male ≤ 1.7 mg/dL<br>Female ≤ 1.5 mg/dL	≤ 2.5
Insulin used to control blood glucose (BG)	Acceptable if BG maintained < 250 mg/dL	Acceptable if BG maintained < 250 mg/dL

HbA1c	≤ 5.7%	No limit
AST/ALT	≤ 3x upper limit	≤ 3x upper limit
Amylase/Lipase	≤ 3x upper limit	≤ 3x upper limit
Hepatitis - evidence of infection	Not acceptable	Not acceptable
Covid-19	No active infection	No active infection
MRSA	Not acceptable	Not acceptable
Rabies	Not acceptable	Not acceptable
SARS	Not acceptable	Not acceptable
Sepsis	Not acceptable	Not acceptable
TB	Not acceptable	Not acceptable
WBC	$< 20 \times 10^9$/L	$< 20 \times 10^9$/L
Blood culture	Negative	Negative
Marijuana usage	If yes, consult on-call PI/Designee	If yes, consult on-call PI/Designee
Alcohol usage	If yes, consult on-call PI/Designee	If yes, consult on-call PI/Designee
Toxicology screen	If positive consult with on-call PI/Designee	If positive consult with on-call PI/Designee
**Organ procurement characteristics**		
Clamp to estimated time of arrest	≤ 12 hrs<br>If 12-15 hrs consult on-call PI/Designee	≤ 15 hrs
Organ damage	No	No
Fatty organ	Maybe	Maybe
Fibrotic organ	Not acceptable	Not acceptable
Calcified organ	Not acceptable	Not acceptable
Edematous organ	Maybe	Maybe
Flush	If not flushed well, consult with on-call PI/Designee	If not flushed well, consult with on-call PI/Designee
Spleen/Duodenum attached	Yes	If not, consult with on-call PI/Designee

Table S2. Impact of donor HbA1c levels on islet outcome and quality

Factors	Non-diabetic (n = 425)	Pre-diabetic (n = 160)	Diabetic (n = 74)	p value		
				**Three groups***	**Non-diabetic vs Pre-diabetic****	**Non-diabetic vs Diabetic****
**Donor characteristics**						
Donor age (years)	44 (31, 52)	50 (41, 55)	52 (45, 58)	**< 0.001**	**< 0.001**	**< 0.001**
Donor BMI (kg/m^2)	29.1 (25.6, 33.4)	29.7 (26.0, 33.1)	31.8 (27.5, 36.5)	**0.005**	0.7	**0.003**
Days in hospital	4.0 (3.0, 6.0)	4.0 (3.0, 7.0)	4.0 (3.0, 7.0)	**0.039**	0.3	0.053
Cold ischemia time (minutes)	425 (348, 575)	421 (353, 498)	415 (338, 525)	0.4	0.6	0.3
Pancreas weight (g)	95 (79, 110)	96 (83, 112)	97 (78, 118)	0.6	0.7	0.6
**Islet isolation outcomes**						
Digestion switch time (minutes)	12.0 (10.5, 14.0)	11.7 (10.0, 13.0)	13.0 (11.8, 15.4)	**< 0.001**	0.3	**< 0.001**
Islet yield outcome (success if islet IEQ > 250,000)				**0.003**	0.7	**0.002**
Success (%)	163 (39%)	55 (36%)	12 (18%)			
Non-success (%)	252 (61%)	99 (64%)	56 (82%)			
Islet purity (high if purity > 80%)				0.15	0.6	0.11
High purity (%)	211 (54%)	73 (49%)	26 (41%)			
Low purity (%)	182 (46%)	77 (51%)	37 (59%)			
Islet recovery post-culture (high if recovery > 75%)				0.8	0.8	0.6
High recovery (%)	205 (51%)	74 (49%)	36 (55%)			
Low recovery (%)	197 (49%)	76 (51%)	30 (45%)			

**Islet quality control parameters**						
Islet viability post-culture (%)	96.0 (93.9, 97.6)	95.8 (93.0, 97.8)	96.3 (94.0, 98.0)	0.7	0.7	0.6
Transplantable islet prep (defined as islet yield ≥ 300,000 IEQ and viability post-culture ≥ 93%)***				**0.022**	0.8	**0.008**
Transplantable (%)	78 (18%)	27 (17%)	4 (5%)			
Suboptimal (%)	347 (82%)	133 (83%)	70 (95%)			
Islet perifusion stimulation index	6.0 (4.0, 9.0)	4.0 (3.0, 6.0)	3.0 (2.0, 6.0)	**0.006**	0.060	0.053
Percent reversal of diabetes in mice	75 (19, 100)	67 (0, 100)	0 (0, 0)	**< 0.001**	0.4	**< 0.001**

The data express as median (Q1, Q3) (Q1 = 25th percentile, Q3 = 75th percentile), n (%)

Non-diabetic, HbA1c < 5.7%; Pre-diabetic, 5.7 % ≤ HbA1c < 6.5%; Diabetic, HbA1c ≥ 6.5%

BMI, body mass index; HbA1c, hemoglobin A1c; IEQ, islet equivalent

* Kruskal-Wallis rank sum test; Pearson's Chi-squared test

** False discovery rate correction for multiple testing

*** The criteria were selected according to the results derived from transplant cohorts in this study (average islet yield > 300,000 IEQ and viability 93%).

Table S3. Summary of islet isolation information from centers covering ~30 years of activity

Publication	Cases	Donors (age/sex)	Pancreas	Yield (IEQ)	Purity/Viability	Function
Diabetol Metab Syndr. 2023 Jul 1;15(1):144	46	N/A, 46 F, 54 M	N/A	N/A	N/A	N/A
Cells. 2022 Jul 29;11(15):2335	82	54.6 years old, 43 F, 39 M	N/A	59,593 ± 56,651	71.5%	SI: 3.85 ± 1.85
Cell Transplant. 2021 Jan-Dec;30: 9636897211052291	55	46 ± 1.6 years old, N/A	6.6 ± 0.3 h	264,323 ± 21,900	78.7 ± 0.9% / 96.0 ± 0.3%	SI: 2.2 ± 0.2
Islets. 2018 Mar 4;10(2):51-59	71	43.9 years old, N/A	CI: 6.96 h	N/A	N/A	N/A
Cell Transplant. 2017 Jan 24;26(1):33-44	119	N/A	N/A	N/A	N/A	N/A
Endocrinology. 2016 Feb;157(2):560-9	142	N/A, 66 F, 76 M	N/A	271,300 ± 17,600	67.7% ± 2.5% / N/A	SI: 11.0 1.0
Diabetes. 2016 Nov;65(11):3418-3428	75	N/A, 22 F, 53 M	N/A	582,370	N/A	N/A
Am J Phys End Metab. 2015 Mar 1;308(5): E362-9	254	44.6 years old, N/A	CI: 8.12 h	353,703	N/A / 94.26%	N/A
Am J Phys Endo Met. 2015 Apr 1;308(7): E592-602	202	42.9 ± 14.1, 74 F, 82 M	CI: 10.1 ± 4.3 h	N/A	81.6 ± 13.0% / 91.2 ± 6.8%	N/A
Transplant Direct. 2015 May;1(4): e14	221	44.2 years old, 58 F, 163 M	N/A	N/A	N/A	N/A
Cell Transplant. 2015;24(9):1879-86	326	44.2 years old, N/A	N/A	N/A	N/A	N/A
Transpl Int. 2014 Sep;27(9):949-55	564	48.8 years old, 225 F, 339 M	CI: 6.19 h	N/A	N/A	N/A

Cell Transplant. 2013;22(12):2323-33	276	N/A	N/A	N/A	N/A	N/A
Transplantation. 2012 Apr 15; 93(7): 693–702	249	45 ± 4.7 years old, N/A	N/A	N/A	N/A	N/A
Proc (Bayl Univ Med Cent) 2010;23(4):341–348	79	42.2 years old, 32 F, 47 M	CI: 5.3 h	N/A	N/A	N/A
Cell Transplant. 2008;17(7):785-92	289	41 years old, N/A	N/A	N/A	75% / N/A	N/A
Transplantation. 2008 Apr 15;85(7):950-5	171	N/A	N/A	223,000 ± 14,000	N/A	N/A
Transplantation. 2006 Oct 15;82(7):983-5	86	N/A, 36 F, 51 M	CI: 8.01 h	356,000	80% / 83%	SI: 5.1
Transplant Proc. 2005 Mar;37(2):1313-4	112	N/A	N/A	N/A	N/A	N/A
Transplantation. 2004 Sep 27;78(6):880-5	114	40.5 years old, 56 F, 58 M	N/A	N/A	61.1% / 94.8%	Basal: 0.173 μU IRI/ng Release high: 7.7 μU IRI/ng
Diabetes. 2003 May;52(5):1143-6	76	45.12 years old, N/A	CI: 7.99 h	463,888	N/A	Basal: 2.6 μU IEQ 90 min Release high: 14.1 μU...
Cell Transplant. 1999 May-Jun;8(3):285-92	14	42.4 years old, N/A	CI: 5.2 h	431,000	N/A	Basal: 0.10 μU IE/min Release high: 0.58 μU...
Cell Transplant. 1999 Nov-Dec;8(6):709-12	80	35 years old, 44 F, 36 M	CI: 4.7 h	173,000, range 10,000-625,000	N/A	Basal: mean 5.1, pg/islet/min. SI: mean 2.8
Transplantation. 1994 Mar 27;57(6):954-8	50	35.5 years old, 21 F, 29 M	CI: 6.3 h	300,000	73% / N/A	N/A

IEQ, islet equivalent; N/A, not available; CI, cold ischemia, SI, stimulation index derived from insulin release assay

Figure S1. Natural history of human islet isolation at City of Hope from 2000 to 2024. A timeline and overview of human islet isolation at the City of Hope National Medical Center from 2004 to 2024 highlights external and internal factors that impacted the isolation process. (a) dissemination of the Edmonton protocol and the Clinical Islet Transplant Consortium standardization isolation practices; (b) implementation and use of a new dissociation enzyme following the discontinuation of Liberase HI; (c) initiation of islet distribution by Islet Cell Resources; initiation of islet distribution by the IIDP; (d) Food and Drug Administration (FDA) approval of clinical islet transplantation as a cell therapy for type 1 diabetes.

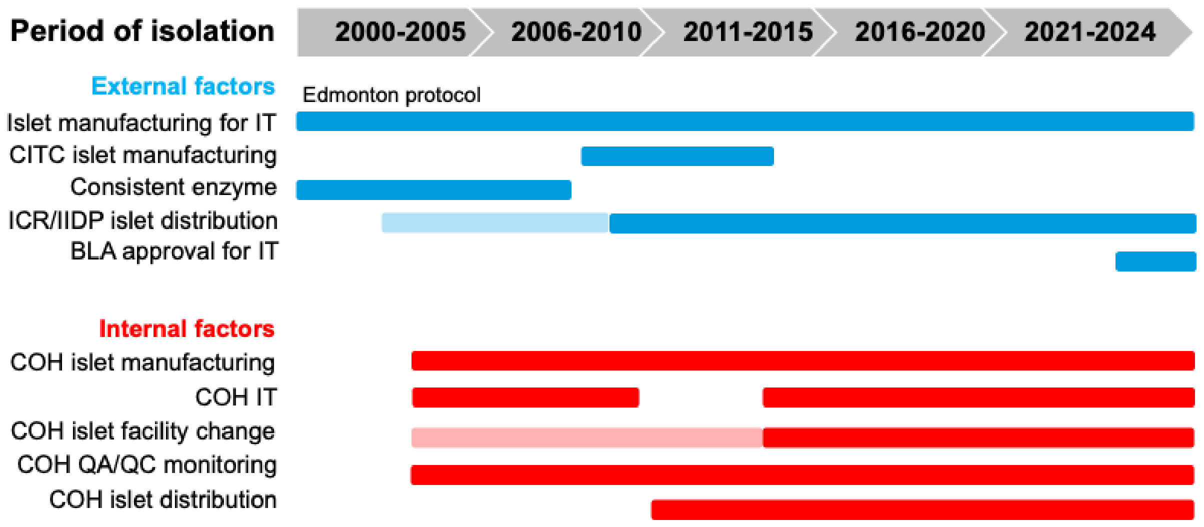

IT, Islet Transplantation; **CITC**, Clinical Islet Transplant Consortium; **ICR**, Islet Cell Resources; **IIDP**, Integrated Islet Distribution Program; **BLA**, Biologics License Application; **COH**, City of Hope; **QA**, Quality Assurance; **QC**, Quality Control
